# Photocatalytic Degradation of Orange G Dye by Using Bismuth Molybdate: Photocatalysis Optimization and Modeling via Definitive Screening Designs

**DOI:** 10.3390/molecules27072309

**Published:** 2022-04-02

**Authors:** Brijesh Kumar Shukla, Shalu Rawat, Mayank Kumar Gautam, Hema Bhandari, Seema Garg, Jiwan Singh

**Affiliations:** 1 Department of Chemistry, Amity Institute of Applied Sciences, Amity University, Sector-125, Noida 201313, India; bshukla2@gmail.com; 2 Department of Environmental Science, Babasaheb Bhimrao Ambedkar University, Lucknow 226025, India; shalurawat200@gmail.com (S.R.); mayankkumar10041998@gmail.com (M.K.G.); 3 Department of Chemistry, Maitreyi College, University of Delhi, Delhi 110021, India; hembrij@gmail.com

**Keywords:** visible light, Orange G, photocatalysis, definitive screening designs, water purification, environmental remediation

## Abstract

In the current study, Bismuth molybdate was synthesized using simple co-precipitation procedure, and their characterization was carried out by various methods such as FT-IR, SEM, and P-XRD. Furthermore, the photocatalytic degradation of Orange G (ORG) dye using synthesized catalyst under visible light irradiation was studied. Response surface Method was used for the optimization of process variables and degradation kinetics evaluated by modeling of experimental data. Based on the experimental design outcomes, the first-order model was proven as a practical correlation between selected factors and response. Further ANOVA analysis has revealed that only two out of six factors have a significant effect on ORG degradation, however ORG concentration and irradiation time indicated the significant effects sequentially. Maximum ORG degradation of approximately 96% was achieved by keeping process parameters in range, such as 1 g L^−1^ loading of catalyst, 50 mg L^−1^ concentration of ORG, 1.4 mol L^−1^ concentration of H_2_O_2_ at pH 7 and a temperature of 30 °C. Kinetics of ORG degradation followed the pseudo first order, and almost complete degradation was achieved within 8 h. The effectiveness of the Bi_2_MoO_6_/H_2_O_2_ photo-Fenton system in degradation reactions is due to the higher number of photo-generated e- available on the catalyst surface as a result of their ability to inhibit recombination of e- and h+ pair.

## 1. Introduction

**Figure 1:**
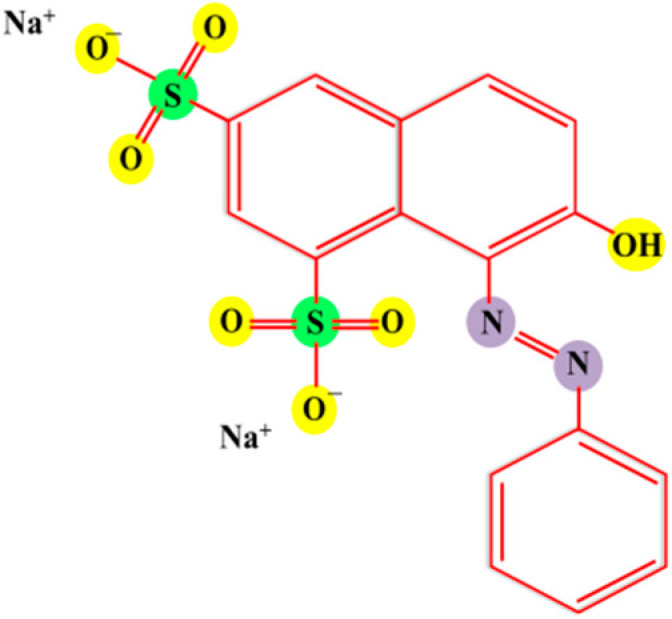
Molecular structure of ORG dye.

Nowadays, environmental issues are a big challenge across the globe and major pollution and global energy demand have created fear amongst humans [1,2,3]. The increasing demand of industrialization force environmental pollution and is certainly the result of urbanization over a period of time, through which an increasing crisis is threatening living things globally. Furthermore, the increasing water crisis is a huge problem and it has been well documented and acclaimed that contaminated water is the foremost source of human loss and mutagenic diseases globally. Wastewater from the dyes industry is a major water pollutant, particularly the material initiatives. Mostly, aromatic-azo-dyes signify 65–75% of the common textile dyes businesses [4] and this segment uses more than 75% of yearly production, projected at around 7 × 10^5^ metric tons [5]. Clearly, a huge tonnage of dyes are polluting water bodies through textile wastes [6]. The main applications of ORG dye is mainly in the color industries, which produce a pH indicator to color keratin, and is also broadly used in the textiles, food [7], and leather industries. ORG is an acid azo dye, and its molecular structure is shown in Figure 1. This dye causes chromosomal damage, and clastogenic activity as inimitable toxic effects [8].Therefore, water conservation and their purification technique should be an absolute priority for researchers fighting this crisis [9,10,11,12,13,14,15,16]. To provide some context, researchers have explored the various methods for removal of ORG, such as adsorption [17], advanced oxidation process (AOP) [18], photo-Fenton process [19], photocatalysis [20,21,22,23,24,25], etc.

Recently, well-known bismuth derived materials have gained attention in the arena of photocatalysis due to their semiconductors feature and the fact that they have a unique structure that can absorb a visible range of light [26,27,28]. Several bismuth derived photocatalysts such as Bi_2_O_3_, BiOX (X = F, Cl, Br and I), Bi_2_WO_6_ BiVO_4_, Bi_2_O_2_CO_3_, and Bi_2_MoO_6_, etc., have been developed for photocatalytic applications [29,30,31,32]. As such, Bismuth molybdate (Bi_x_MoyO_z_) has been well recognized as a catalyst for the mineralization of pollutants under visible-light irradiation due to features such as its structure, non-toxicity, and effectiveness towards visible light spectrum [33]. 

Many studies have been conducted to improve the photocatalytic activity of bismuth molybdate by tuning its structure, however, research work is still going on to enhance their Photocatalytic activity due to issue of electron-hole recombination. Therefore, the photo-Fenton method is required to optimize the process parameters and get the optimum catalyst activity by using statistical methods, to improve the absorption of light towards photo-degradation [34,35].

In a descriptive article, researchers have explained the drawback of one factor at a time (OFAT) designs and expressed the view that they favor factorial design (FD) over OFAT designs [36]. The FD is certainly superior to the OFAT design in a number of significant ways, and furthermore, if the number of study variables is large then factional factorial design (FFD) is preferred. For example, an FFD resolution IV, can estimate two-factor interactions and is also less sensitive to outliers than OFAT designs. FFD have many advantages over OFAT and are able to understand the main effects and interaction effects of process variables and their magnitude in fewer experiments [37].

In the present study, bismuth molybdate material was synthesized using a simple co-precipitation process and their photocatalytic activity was examined for the removal of ORG dye under visible-light illumination. Moreover, the process conditions of photocatalysis were optimized by a statistical method using Definitive Screening designs, and a response surface method.

The core purpose of the current study:

(1) To evaluate the application of the bismuth molybdate materials towards the photocatalytic degradation of ORG dye;
(2) To estimate the significant outcome of six process parameters, such as photocatalyst loading, dye concentration, irradiation time, temperature, pH, and the role of H_2_O_2_, as added for electron (e^−^) scavenger;
(3) The modeling and optimization of process variables for Photocatalytic degradation by statistical method; Definitive Screening design, and a response surface designs method.

Various methods have been used so for to synthesize the Bi_2_MoO_6_ photocatalyst, such as the hydrothermal process [38] and solvothermal process [39]. In order to improve the photocatalytic activity of Bi_2_MoO_6,_ a few authors have modified it using different techniques such as the preparation of composites with Cds QDs [40] and by absorbing C_60_ on the surface of Bi_2_MoO_6_ [41] etc. In comparison with these reports, this study shows a successful synthesis of bismuth molybdate photocatalysis by a facile co-precipitation method without any further modification and heat treatment. Hence the synthesis is economical and, due to the lack of participation of any toxic solvent in the process, this method does not cause environmental pollution. The catalyst is visible light-active, which makes it efficient to harness the visible region of natural solar light that is around 43% of the solar spectrum, therefore it can replace the previously reported UV-light active photocatalysts such as zinc oxide [42] and titanium dioxide [43], which have been in use until now. Furthermore, the use of statistical tools to optimize the degradation process parameters, instead of one parameter at a time, reduced the time consumption and provided information about the complex degradation system.

## 2. Materials and Methods

### 2.1. Materials for Synthesis

Bismuth Nitrate (Bi(NO_3_)_3_.5H_2_O), Sodium molybdate ((NaMoO_4_.2H_2_O) Sodium hydroxide, Nitric acid, and H_2_O_2_ were purchased from Sigma-Aldrich (New Delhi, India. ORG dye was purchased from Spectochem, New Delhi, India. The available chemicals, and reagents were lab grade and used without further refinement.

### 2.2. Synthesis of Bismuth Molybdate

Bismuth molybdate was synthesized by dissolving1.4 mmol of Bi (NO_3_)_3_.5H_2_O in 1.0 mL of nitric acid and 5 mL of water using magnetic stirring. Then, 5 mL aqueous solution of 0.70 mmol of NaMoO_4_·2H_2_O was added at room temperature. The pH of the suspension mass was adjusted to six–seven by adding 10% aqueous sodium hydroxide solution at room temperature. After, the precipitated solid materials were kept at 60 °C for 4 h. Then, the reaction mass was allowed to cool down at room temperature and the precipitated material was filtered and washed several times with water, followed by ethanol. The sample was dried at 90 °C for 10 h in a vacuum oven and dried material was crushed to make a uniform powder prior to use for photocatalysis applications.

### 2.3. Instrumentation

A portable pH-meter (model 371 Water Analyzer, Systronics, Ahmedabad, India) was used for pH adjustment at the required values. The absorption was recorded in the range of 200 nm to 700 nm using a UV-visible spectrophotometer (117 Systronics, Ahmedabad, India). Morphological details of the catalyst evaluated by SEM (JSM 4490, JEOL, Tokyo, Japan, equipped with EDS). FTIR (NICOLET 6700, Thermo Fisher Scientific, Waltham, MA, USA) had been used for the functional group identification. The X-ray diffraction was recorded by D8 Advance Eco (Bruker, Ettlingen, Germany) using Cu k_α_ radiation (λ = 0.15 nm) at room temperature in the range of 10° to 80° at a 2θ angle 

### 2.4. Photocatalytic Measurements

The experiments relevant to the degradation of ORG dye were performed in a closed box apparatus equipped with a tungsten lamp (100 W), having wavelengths of 400–800 nm of the visible range of spectra. The UV-DRS of the catalyst reflected that its absorption falls in the visible range. The photon flux was determined to be 500 lux. ORG dye aqueous solutions (100 mL) with an appropriate amount of catalyst, H_2_O_2_ and obtained solution was transferred into a double walled cylindrical glass reactor equipped with water circulation and a magnetic stirrer. The pH of the reaction mass was maintained by adding a sufficient amount of dilute H_2_SO_4_ or NaOH aqueous solutions and all the process conditions and quantities specified in the experimental design (Table 1). The adsorption and desorption stability of the dye on the surface of the photocatalyst was achieved by keeping the suspension in the dark for 30 min prior to illumination. Then, 4 mL of aliquots were taken from the reaction mass at hourly intervals and were centrifuged, followed by micron filtration by Whatman filter paper grade 1, with pore size 11 μm (purchased from Axiva Sichem Biotech (New Delhi, India) in order to eliminate the suspended particles prior to UV absorption reading. The ORG dye concentration was estimated by measuring the absorbance at the wavelength of 475 nm using a UV–Vis spectrophotometer (Systronics 117).

### 2.5. Experimental Design and Data Analysis

Six factors selected as independent operating variables and studied factors are catalyst Loading; (A), ORG dye concentration (B), H_2_O_2_ concentration (C), irradiation time (D), Temperature (E), and pH (F). A Definitive Screening Design, a response surface method, was used for the experimental design for the optimization of photocatalytic degradation of the ORG dye and considered six operating variables (n = 6) with their high (+1) and low (−1) level (−). The total number of experiments was 13, and consisted of 10 factorial points, and 3 central points, as shown in Table 1. The experiments presented with actual values at low and high levels with a center point, and were all performed in triplicate. By considering the range of ORG dye concentration in industrial wastewaters to be 0.5–100 mg L^−1^, the concentration range of 20 (−1) to 100 (+1) mg L^−1^ was selected for the study range based on kinetics and real wastewater concentration. In addition, H_2_O_2_ concentration in the range of 0.6 mol L^−1^ (−1) to 1.4 mol L^−1^ (+1) was selected to counter the acceleration of the e^−^/h^+^ recombination effect, which may occur due to the presence of other organic components in the real effluent. Moreover, all the six factors and their study ranges within the design summary are summarized in Table 1. The efficiency of the degradation was estimated by determining the degradation % of ORG after a specified time of irradiation, according to Equation (1):

(1) $$\%\;\mathrm{Degradation}\;\mathrm{ORG}=\frac{C_{0}-C_{t}}{C_{0}}\times 100$$

where, C_0_ and C_t_ are the ORG dye concentration before and after photocatalysis treatment, respectively.

The default analysis in Design Expert begins with a full quadratic polynomial and recommendation to decrease the number of terms to a unaliased subset and to have an option to select terms either manually or by using the automatic selection algorithm to produce a meaningless model. A first order least square linear equation (Equation (2)) was used to fit the experimental run of the design as follows:

(2) $$Y=\beta_{0}+\beta_{1}X_{1}+\cdots +\beta_{kk}+\ldots +\beta_{12}\ldots kX_{1}X_{2}\ldots X_{k}$$

where *Y* denotes the response, i.e., ORG dye removal efficiency; *X*_1_, *X*_2_, and *X_k_* are the considered independent variables; *β*_0_, *β*_1_, *β*_12,_ *β_kk_* and *β*_12_ are the coefficients for the first order and the interaction terms, respectively.

The adequacy of the model was tested by the analysis of variance (ANOVA) and all the assumptions were analyzed for the meaningful results. Further violation of regression assumption was checked by several diagnostic methods, which included a graphical illustration of predicted vs. experimental values, to check the normality of the residuals by a normal probability plot of the studentized residuals, to look continually for errors made by studentized residuals vs. predicted plot, to check the outlier by cook distance, and the need of power transformations were checked by the Box-Cox plot [44].

The model accuracy and fitness statistics of the suggested model from the Response surface model were estimated by the F-statistic, the R-Square value (R^2^), adjusted R-Square value and the predicted R-Square value (R^2^ pred) for the parameters examined. The probability value of 0.05 with a 95% confidence level has been used to determine the statistical significance of all model coefficients. The magnitude of effects of the individual process factors on ORG removal were illustrated using 3D response surface plots. Finally, a desirability function was used to set the optimum process conditions to maximize ORG dye degradation. The region that maximizes the desirability function was determined using numerical optimization. The trial version of the Design-Expert 13 (StatEase Inc., Minneapolis, MN, USA) application was used during the experimental design, statistical analysis, contour plots, and numerical optimization.

## 3. Results and Discussion

### 3.1. UV-Visible, SEM, FT-IR, and XRD Characterization of Bi_2_MoO_6_

**Figure 2:**
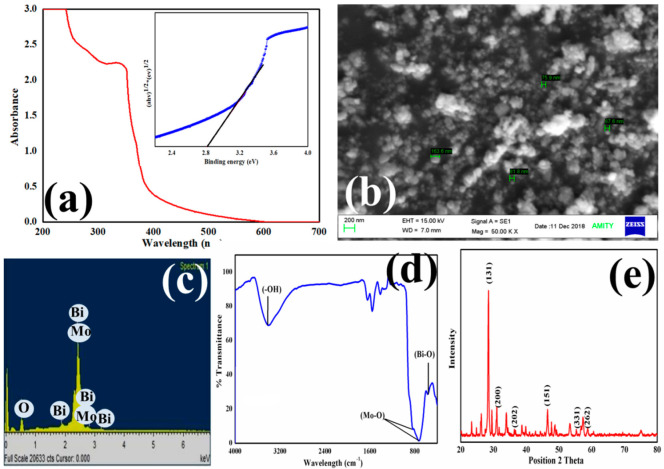
(**a**) UV-Visible analysis (Tauc’s plot inset) (**b**) SEM, (**c**) EDS, (**d**) FTIR and (**e**) XRD analysis of the photocatalyst.

The UV-Visible scan of the photocatalyst is shown in Figure 2a and it shows the absorption of the spectrum in the range of 200 to 500 nm. The value of binding energy was found to be 2.8 eV by the Tauc’s plot shown inset. The band gap energy was in accordance with the previously reported work on bismuth molybdate [45]. The Bi_2_MoO_6_ intrinsic energy gap transition from valence band to conduction band of O 2p orbitals are mainly from the Mo 4d orbitals in the octahedrons of MoO_6_, whereas the absorption edge shape is attributed to the secondary Bi 6p [46]. The morphology of Bi_2_MoO_6_ materials was examined using SEM images. The SEM images of Bi_2_MoO_6_ reveal the rod-like structure imbedded with spherical particle as shown in Figure 2b. The relative EDAX mapping (Figure 2c) was further conducted, which confirmed that the structure is composed of Bi, Mo, and O demonstrating a uniform dispersion of Bi_2_MoO_6_. The FT-IR spectra of Bi_2_MoO_6_ are depicted in Figure 2d. The high frequency region peak, placed at 3404 cm^−1^, are designated to OH groups in the Bi_2_MoO_6_, and further the lower wavelength region peaks from 500 to 1000 cm^−1^ corresponding to the presence of metal oxide bonds [47], and characteristic peaks at 838 cm^−1^, 730 cm^−1^, and 567 cm^−1^ are indicted to the stretching vibration of Mo-O and Bi-O, which reveal vibration in the crystal. A characteristic peak at around 838 cm^−1^ is reported to correspond with the asymmetric vibration stretch of MoO_6,_ which is related to the apical oxygen atom vibrations, and the peak at 730 cm^−1^ corresponds to the MoO_6_ asymmetric stretch, which comprises the central oxygen atom vibrations [48]. Two characteristic peaks at 838 cm^−1^ and 730 cm^−1^ are close to the symmetric stretching of MoO_6_ vibration bonds of the apical oxygen atoms. The Powder-XRD analytical technique is the best known technique and has been used for decades to analyze the crystallinity of materials. Figure 2e depicted the bismuth molybdate PXRD diffraction pattern and more intense diffraction 2 theta values of 28.30°, 32.54°, 35.95°, 46.68°, and 55.56 and their corresponding planes (131), (200), (151), (202), and (331) of the relative diffraction pattern, well accorded with the JCPDS (Number: 21-0102) and referring to the orthorhombic structure of Bi_2_MoO_6_ [49,50]. 

### 3.2. Photocatalytic Degradation of ORG

#### 3.2.1. Preliminary Experiments

Preliminary photocatalysis experiments were performed to investigate probable parameters such as catalyst loading, reaction pH, and dye concentration to check the impact on degradation of dye prior to the experimental design. However, direct photocatalytic degradation resulted in ~25% removal of ORG dye. Moreover, the impact of H_2_O_2_ was also evaluated for the photocatalytic performance of ORG dye, and the photocatalytic degradation of ORG dye was enhanced significantly, leading to 50% removal due to its e^−^ scavenger properties, inhibiting electron-hole recombination efficiently, leading to enhanced degradation of ORG dye.

#### 3.2.2. Process Parameters Optimization with Statistical Modeling for ORG Photocatalytic Degradation

The experimental and predicted values of the photocatalytic degradation of ORG dye are depicted in Table 1. Post elimination of the non-significant model term using the automatic selection algorithm of a design expert; a first order linear model was established to define the empirical relations between the coded variables and the % degradation of ORG dye response according to the following equation:

(3) % Degradation of ORG dye=+79.18−11.87×B+7.98×D      

ANOVA analysis was performed (results are shown in Table 2) to evaluate the statistically significant model terms and the linear model was established, including only significant model terms that were best fitting, and model hierarchy for the prediction of ORG dye degradation. 

From ANOVA analysis, a *p* value of 0.0013 and a corresponding F value of 15.26 suggest a highly significant model and there is only a 0.135% chance that an F-value this large could occur due to noise. *p*-values less than 0.05 indicate model terms are significant. In this case B (ORG dye concentration, and D (irradiation time) are significant model terms. Values greater than 0.10 indicate the model terms are non-significant. In this case there are many insignificant model terms (not included to support hierarchy), and further model reductions have been conducted to obtain an improved model. On the other hand, the *p* value of the respective model terms indicates a suitable model and is well suited in the experimental data prediction. The coefficient of R-Square value (R^2^) of the regression model is 0.7722, indicating that more than 77.22% of the data deviance can be described by the model.

**Figure 3:**
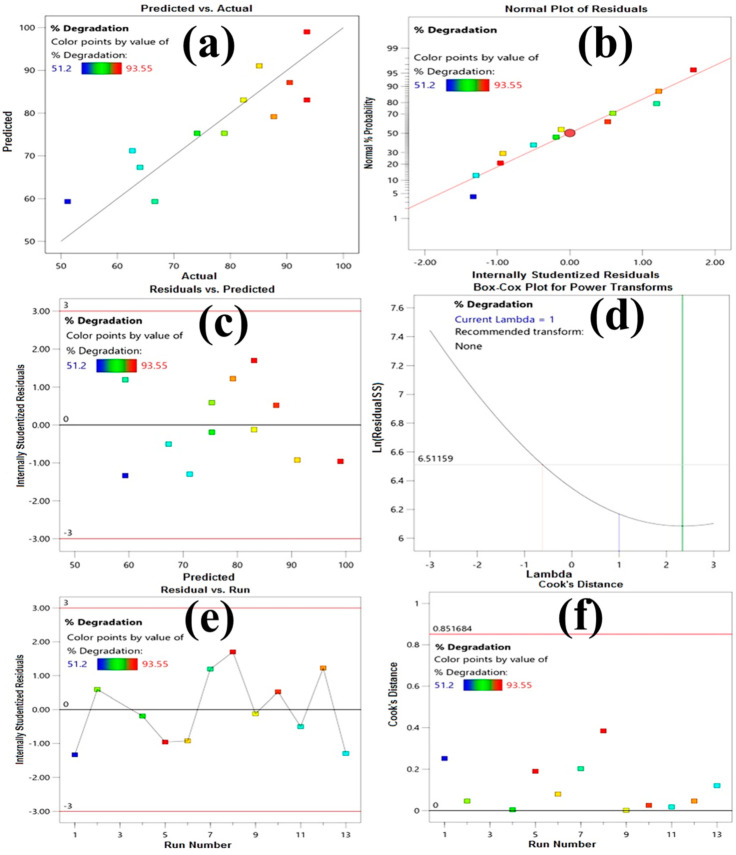
Different Diagnostics Plots for model adequacy: (**a**) predicted vs. actual; (**b**) normal probability; (**c**) internally studentized residuals vs. predicted values; (**d**) Box-Cox; (**e**) Residual vs. Run; and (**f**) Cook’s Distance.

Likewise, regarding the value of the Adjusted R-Square (adj. R^2^) value of 0.7216 and the predicted R-Square (Pre R^2^) value of 0.5931, the difference is less than 0.20, and there is reasonable agreement as to the predictability of the model. Furthermore, several graphical diagnostic tools were used to analyze model adequacy, i.e., predicted vs. actual-values (Figure 3a), normal-probability plot (Figure 3b), residual vs. predicted-values (Figure 3c), the cook distance (Figure 3f), Box Cox plot (Figure 3d), and the residual vs. run (Figure 3e) [51,52,53] and indicated the suitability of the model.

The experimental values (Y_exp._) and model calculated value, i.e., predicted value (Y_cal._) (Figure 3a) again indicate the good agreement and fitting ability of the recommended model. Furthermore, the internally studentized residual values in Figure 3b, which are close to a straight line, added to the model’s suitability to define the correlation between the considered factors and the degradation response. The well distribution of residual values between −2 and +2 with random scattering, as depicted in Figure 3c, also revealed a similar prediction. The Box–Cox plot (Figure 3d) shows that there is no transformation for the response, indicating that the data is normally distributed and the model can be predicted well [54,55,56]. All the independent variables and their interaction effects are highly significant factors with *p* values of <0.05. The influence of each factor on the degradation was estimated agreeing to the first order linear regression Equation (3). According to regression analysis, ORG dye concentration (11.87%), and irradiation time (7.98%) demonstrated the maximum effects on photocatalytic degradation effectiveness, whereas other factors did not show significant effects within the studied range on photocatalytic degradation efficiency and were not included for analysis for model hierarchy and good predictability of model. The first order Equation (3), obtained from regression analysis, indicates positive or negative effects of the independent variable by their positive or negative signs [57]. ORG dye concentration (B) showed the existence the main negative effects, showing that degradation of dye removal is reduced by increasing the dye concentration from the lower to the higher side within the study range. The excessive increase in dye concentration overcomes the number of reactive radicals generated, and therefore the excess amount of dye remains un-degraded, and in higher concentrations the dye molecules also tend to adsorbe over the surface of the catalyst, which masks their active sites [58]. Additionally the increase in dye concentration results in more absorption of the light by the dye molecules, which hinders the penetration of light to the catalyst surface [59]. Due to all these reasons, the degradation of dye decreases with increasing concentration of dye. In comparison, irradiation time (D) has been shown to have the maximum positive effect, indicating that degradation increases with increasing irradiation time.

**Figure 4:**
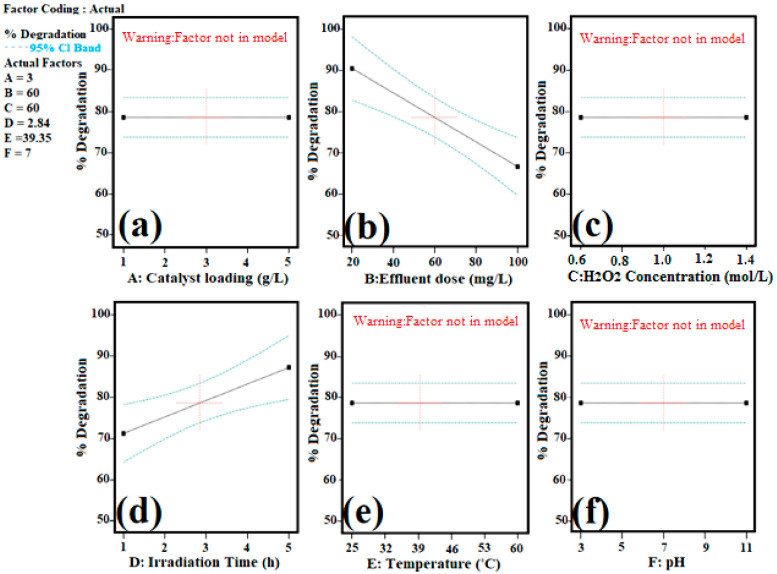
Main effect plot % degradation vs. process variable: (**a**) catalyst loading; (**b**) ORG concentration; (**c**) H_2_O_2_ concentration; (**d**) Time of irradiation; (**e**) Temperature; and (**f**) pH.

**Figure 5:**
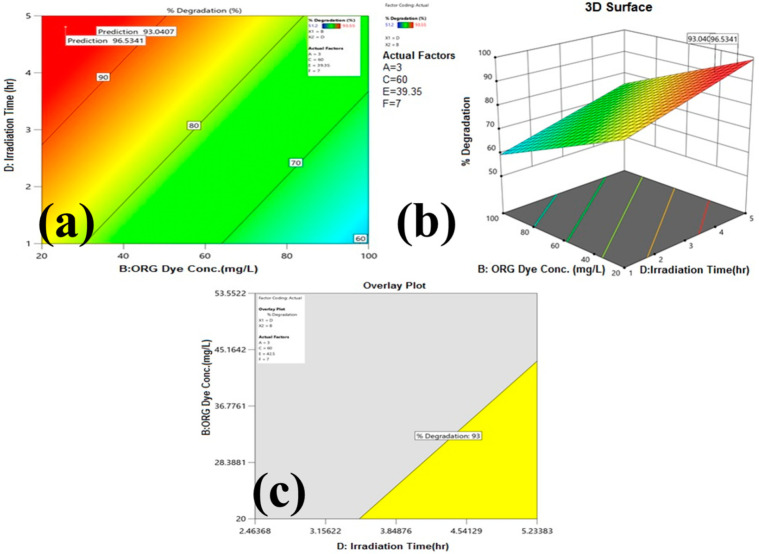
Contour plot and Three-dimensional response surface plots for ORG dye photocatalytic degradation: (**a**) Contour plot-ORG concentration vs. time of irradiation; (**b**) 3D Response surface plots-ORG concentration vs. time of irradiation; (**c**) overlay plot for design space (yellow region in the overlay plot is the operating range for significant factors). Several researchers used Orange G dye as a model pollutant for photocatalytic degradation comparative results, which are summarized in Table 3.

The magnitude of the different process parameters’ effects on ORG dye degradation were illustrated in graphical representation in Figure 4a–f and factors having a significant effect on degradation were visualized by contour (Figure 5a) and 3D response surface plot (Figure 5b).

Based on preliminary experimentation observations, the effectiveness of the degradation rate of ORG dye was increased with an increase in catalyst dosing up to a certain extent, above which the degradation rate was diminished, and with a further increase in catalyst dosing, the degradation began to decrease. It was most likely due to opaqueness of the reaction mixture caused by the increase in the amount of catalyst, which hinders the proper penetration of light in the reaction mixture and, as such, reduces the degradation efficiency of the system [60]. Secondly, on increasing the catalyst concentration, catalyst particles also may tend to agglomerate, which causes overlapping in the active sites so less reactive radicals were generated resulting in a decrease in degradation efficiency [61]. However, the efficiency of degradation remains more than 65% in the range of study, irrespective of process parameters.

Meetani et al. [62] explored the effect of pH on the degradation of ORG dye by using a mixed oxide catalyst and explained that the degradation decreased from 50% to 30% by lowering the pH value from five to three. This was due to an excess of H^+^ being offered for deposition to cover the surface of the catalyst, consequently inhibiting the photo-excitation of catalyst active sites, thereby inhibiting the formation of free radicals.

Moreover, similar observations were made with higher pH as compared to the natural pH, and due to higher pH values, -OH ions will be deposited on the catalyst surface sooner, henceforth inhibiting the complete effectiveness of the photocatalyst [62]. ORG dye is an anionic dye, and containing a sulfuric group it acquires a negative charge on it in an aqueous medium, as shown in Figure 1. The absorption of OH- ions on the catalyst increases the pH of the medium and make its surface negatively charged, which repels the dye molecules with same electrical charge, and therefore, the interaction between catalyst and dye molecule in alkaline pH is reduced and the degradation efficiency decreases [63].

However, Ou et al. [64] successfully overcame the problem of the narrow pH range for degradation of ORG by FeVO_4_/H_2_O_2_ systems and enhanced the application range from 4.0 to 9.0 from neutral pH. Similarly, the current study explored the wide pH range from three to eleven for degradation of ORG dye with a Bi_2_MoO_6_/H_2_O_2_ catalyst system and did not show any significant negative effect across the wide pH range, as illustrated in the graphical presentation shown in Figure 4f. This advanced the catalyst’s application in real-world industrial effluent without changing the pH of the degrading solution.

The efficiency of degradation is reduced by increasing ORG dye, as the available photo-generated h^+^ becomes the controlling factor for degradation, considering the degradation is a surface reaction [65].

In comparison, based on preliminary experimental results, it has been shown that H_2_O_2_ concentration has a positive effect on the degradation efficacy. This trend can be described by the ability of H_2_O_2_ to act as an effective electron scavenger [66,67], inhibiting the recombination of electron–hole pairs. Instead of H_2_O_2_ being moderately adsorbed, it has the capability to form oxidizing radicals (*) via e^−^ reaction, resulting in the photo-oxidation of ORG to some extent [67].

The numerical optimization of the process parameters using a desirability function was studied by design expert software to find the particular design point that maximizes the % degradation. The best optimized conditions for process parameters need to be set as follows to get maximum degradation efficiency: catalyst loading of 1g L^−1^, ORG concentration of 20 mg L^−1^, H_2_O_2_ = 1.4 mol L^−1^, irradiation time= 5 h, temperature= 25 °C, and pH = 7. However, the adsorption percentage of dye on the photocatalyst surface after 30 min in the dark varied from 12 to 36% for different process conditions, as depicted in Table 1. The difference in the adsorption percentage can be attributed to the nature of the electrostatic forces between dye and the surface of the catalyst, and this is due to varied photocatalytic process conditions such as catalyst loading, dye concentration, pH etc.

Due to the complex structure of dye degradation, kinetics in visible light is slow and it takes 5 h to complete the reaction. However, more than 65% dye degradation was completed within 60 min, later on; the rate of degradation was very slow maybe due to the surface kinetics of the catalyst. The reaction time of this study, however, is less than some of the previously reported photocatalytic degradation studies of dyes under visible light irradiation [20,21]. Furthermore, keeping the same range of process parameters to get maximum degradation generated the overlay plot as shown in Figure 5c for design space, and an overlay plot that has a yellow region is the region for optimum photocatalytic degradation.

**Table 3:** Comparative results of ORG dye degradation by various catalyst.

Catalyst	Synthesis Method	ORG Dye (mg/L)	Light Sources	Degradation	References
N-doped TiO_2_	hydrolysis and calcination of tetrabutyl titanate	25	Visible	96.29%	[68]
ZnO/HS	precipitation and calcination	50	Solar UV	49.78% 94.36%	[69]
Sn(IV)/TiO_2_/activated carbon	Dip-coating technique with TiO_2_ sol–gel	50	UV	99.1%	[70]
ZnO/activated carbon	Carbonization and Precipitation	20 mg	Visible UV	95% 90%	[20]
Bi_2_MoO_6_	Co-precipitation	20–100 mg	Visible	98%	This work

#### 3.2.3. Model Validation, Kinetic and Isotherm Studies

**Figure 6:**
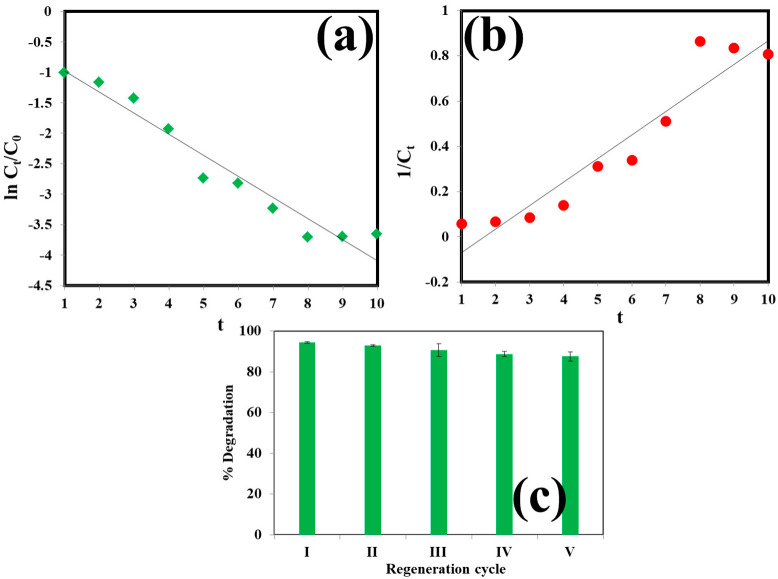
(**a**) Pseudo-first-order and (**b**) pseudo-second-order kinetics plots of ORG dye photocatalytic degradation under optimized conditions (C_cat_ = 1 g L^−1^, C_org_ = 50 mg L^−1^, pH = 7, irradiation time-10 h, temperature−25 °C, H_2_O_2_ = 1.4 mol L^−1^). (**c**) Reusability test of Bi_2_MoO_6_ catalyst.

To check the suitability of the model for forecasting the maximum ORG dye degradation, validation trials were performed using the optimized conditions, except for the irradiation time, i.e., specified 10 h. The validation experiments were tested by the application of pseudo-first-order and pseudo-second-order kinetics, whose linear equations are represented in Equations (4) and (5), respectively,

(4) ln(CtC0)=−k1t

(5) 1Ct=(1C0)+k2t

where *C*_0_ and *C_t_* are the initial concentration and residual concentration after time t of the pollutants, respectively, and *k*_1_ and *k*_2_ are the rate constants for pseudo-first order and pseudo-second order kinetics, individually. Their values were determined from the slope and intercept of their respective plots, as depicted in Figure 6. Under the optimized conditions, pseudo-first-order kinetics was fitted to the removal study with a reaction rate constant of *k*_1_ = 0.345 min^−1^ and an R^2^ value of 0.945, while the values of *k*_2_ and R^2^ for pseudo-second-order kinetics were found to be 0.103 and 0.901, respectively. 

### 3.3. Reusability of the Catalyst

In order to determine the reusability of the prepared catalyst it was recycled for five cycles. The recycling process was carried out at the optimized conditions (i.e., catalyst loading of 1 g L^−1^, ORG concentration of 20 mg L^−1^, H_2_O_2_ = 1.4 mol L^−1^, irradiation time = 5 h, temperature = 25 °C, and pH = 7). After using the catalyst in the first cycle, it was separated from the reaction solution by centrifugation and the recovered catalyst was washed with distilled water, dried in a hot air oven and used in the next cycle. Figure 6c shows the result of catalyst recycling. It shows that the ORG dye degradation was slightly decreased from 94.1% in first cycle to 87.9% in the last cycle. The decrease in degradation efficiency may be due to the adsorption of dye molecules or degradation of products over the catalyst surface, and it also may occur due to the loss of some catalysts in the recycling process. These results show that the synthesized Bi_2_MoO_6_ photocatalyst can be used many times for the dye degradation process.

### 3.4. Mechanism of Orange G Dye Degradation

There are two pathways for the degradation of organic contaminant in a Photocatalytic method. The first one is the irradiation of visible light, which excites the catalyst to generate electron and hole pairs (e^−^/h^+^) in the conduction band and valence band of the catalyst. Electrons present in the conduction band attack the molecular oxygen to produce a reactive oxygen species; additionally, holes (h^+^) generated in the valence band directly form reactive oxygen species by interacting with water. These produce a reactive species of oxygen (OH• and $•O_{2}^{-}$) that initiates the degradation of ORG dye [71,72]. The aforesaid mechanism can be described on the basis of following reactions [73]:

(6) $${\mathrm{Bi}}_{2}{\mathrm{MoO}}_{6}+\mathrm{hv}\to {\mathrm{Bi}}_{2}{\mathrm{MoO}}_{6}\left(e^{-}+h^{+}\right)$$

(7) $$e^{-}+O_{2}\to •O_{2}^{-}$$

(8) $$h^{+}+{\mathrm{OH}}^{-}\to \mathrm{OH}•$$

(9) $$\;•O_{2}^{-}/\mathrm{OH}•+\mathrm{ORG}\to {\mathrm{CO}}_{2}+H_{2}O+\mathrm{intermediates}\;$$

## 4. Conclusions

The Bi_2_MoO_6_ catalyst was synthesized by a simple co-precipitation procedure and the photocatalytic degradation kinetics of ORG dye was explored in detail in the presence of synthesized materials. A reduced response surface method (definitive screening design) was used effectively to optimize significant operational factors to maximize ORG removal. All six factors were investigated (i.e., ORG concentration and catalyst loading, irradiation time, concentration of H_2_O_2_, and pH) for photocatalytic degradation efficiency. However, the concentration of ORG and irradiation time were by far the more critical process parameters, with the concentration of ORG = 20 mgL^−1^ and irradiation time = 5 h being the improved conditions and dye degradation achieving about 98%. However, H_2_O_2_ showed an increased effectiveness in the photocatalytic degradation of ORG dye due to the reduced recombination rate of photo-generated e^−^–h^+^ pairs. The results show that the definitive screening design is a great tool for improving the degradation of ORG dye efficiency by optimizing the conditions of process parameters. Thus the synthesized Bi_2_MoO_6_ was found to be an efficient photocatalyst with a narrower band gap than the most widely used photocatalyst such as TiO_2._ The catalyst was successfully synthesized as its characterizations are in agreement with the previous reported work; however, the co-precipitation method for the synthesis of Bi_2_MoO_6_ photocatalyst was found to be better than other methods in terms of economical input, complexity and easy handling.

## Figures and Tables

**Table 1:** Design summary, Factors and their Ranges with Response, and Design sheet with experimental data and their respective predicted value.

Design Summary									
**File version**				13.0.2.0					
**Study type**				Response Surface		Subtype		Randomized	
**Design Type**				Definitive Screening		Runs		13	
**Design model**				Reduced Quadratic		Blocks		No Blocks	
**Factors and Response**									
**Factors**		**Name**		**Units**	**Type**	**Min**	**Max**	**Coded low**	**Coded high**
**A**		Catalyst Loading		g/L	Numeric	1	5	−1	+1
**B**		Effluent Dose		mg/L	Numeric	20	100	−1	+1
**C**		H_2_O_2_		Mole	Numeric	0.6	1.4	−1	+1
**D**		Irradiation Time		H	Numeric	1	5	−1	+1
**E**		Temperature		°C	Numeric	25	60	−1	+1
**F**		pH			Numeric	3	11	−1	+1
**Response**		**Name**		**Units**	**Observations**	**Min**	**Max**	**Mean**	**SD**
**R1**		% Degradation		%	13	51.2	93.55	77.65	13.22
**Design Sheet, Response and model prediction**									
		**F 1**	**F 2**	**F 3**	**F 4**	**F 5**	**F 6**	**Degradation**	
**Std**	**Run**	**A: Catalyst Loading**	**B:** **Effluent Dose**	**C: H_2_O_2_**	**D:** **Irradiation Time**	**E: Temperature**	**F: pH**	**Experimental**	**Predicted**
		**g L^−1^**	**mg L^−1^**	**mol L^−1^**	**H**	**°C**		**%**	**%**
9	1	5	100	1.4	1	42.5	3	51.2	59.33
1	2	3	100	1.4	5	60	11	78.93	75.29
10	3	1	20	0.6	5	42.5	11	79.06	NA
6	4	1	100	1	5	25	3	74.12	75.29
11	5	5	20	1.4	5	25	7	93.55	99.03
8	6	1	20	1.4	3	60	3	85.12	91.05
12	7	1	100	0.6	1	60	7	66.63	59.33
5	8	5	20	1	1	60	11	93.55	83.08
2	9	3	20	0.6	1	25	3	82.34	83.08
3	10	5	60	0.6	5	60	3	90.52	87.16
7	11	5	100	0.6	3	25	11	64	67.31
13	12	3	60	1	3	42.5	7	87.71	79.18
4	13	1	60	1.4	1	25	11	62.66	71.2

**Table 2:** ANOVA table, Fit Statistics, and Coefficients in Terms of Coded Factors.

ANOVA for Reduced Linear Model						
**Response 1: % Degradation of ORG dye**						
**Source**	**Sum of Squares**	**Df**	**Mean Square**	**F-Value**	***p*-Value**	
**Model**	1618.74	2	809.37	15.26	0.0013	Significant
B- Effluent Dose	1237.98	1	1237.98	23.33	0.0009	
D- Irradiation Time	559.01	1	559.01	10.54	0.0101	
**Residual**	477.5	9	53.06			
**Cor Total**	2096.24	11				
**Fit Statistics**						
**SD**	7.28	**R^2^**			0.7722	
**Mean**	77.53	**Adjusted R^2^**			0.7216	
**C.V. %**	9.4	**Predicted R^2^**			0.5931	
		**Adeq Precision**			10.8995	
**Coefficients in Terms of Coded Factors**						
**Factor**	**Coefficient Estimate**	**df**	**Standard Error**	**95% CI Low**	**95% CI High**	**VIF**
Intercept	79.18	1	2.12	74.37	83.99	
B-Effluent Dose	−11.87	1	2.46	−17.43	−6.31	1.01
D-Time for Irradiation	7.98	1	2.46	2.42	13.54	1.01

## Data Availability

Not applicable.

## Sample Availability

Samples of the compounds are available from the authors.
